# Beyond Obesity

**DOI:** 10.3390/medsci13030176

**Published:** 2025-09-04

**Authors:** George A. Bray, Donna H. Ryan

**Affiliations:** Pennington Biomedical Research Center, Baton Rouge, LA 70808, USA; donna.ryan@pbrc.edu

**Keywords:** clinical obesity, body mass index (BMI), central fat, stigma of obesity

## Abstract

Diagnosis of clinical obesity has been highlighted by the recent publication from a Commission Report in *The Lancet*, suggesting the addition of a new diagnostic category, “Preclinical Obesity,” to the already existing ones. Diagnostic criteria for obesity began in the first half of the 20th century, when life insurance companies provided information tables of ideal body weight levels and/or desirable body weight levels based on actuarial associations with mortality. This was replaced by the body mass index or BMI in the third quarter of the 20th century. This tool documented the epidemic of obesity in the US in the last three decades of the 20th century. The recognition of the importance of fat distribution, pioneered by the work of Jean Vague in France, provided a new understanding of obesity. The limitations of BMI and the availability of effective new treatments have heightened the need for new diagnostic guidelines. Obesity represents an increase in body fat and an alteration in its distribution and function. But at the same time, obesity is a stigmatized word and a pejorative term. This communication discusses ways to better diagnose the increase in body fat and its abnormal distribution. We ask whether there is an alternative word to replace obesity and suggest that adiposity or healthy weight could be options.

## 1. The Recognition of Clinical Obesity

Obesity has recently become prominent due to the publication of two reports. The first was from a group of international experts that *The Lancet* commissioned to “establish objective criteria for diagnosis of obesity, while aiding clinical decision making and prioritization of therapeutic interventions and public health strategies [1].” In this detailed review, the group introduced the concept of both clinical obesity and preclinical obesity (see Table 1). Both are characterized by excess body fat. The report defines clinical obesity by the presence of organ or tissue dysfunction and/or impaired functional capacity for activities of daily living in addition to excess body fat. Individuals without such symptoms or evidence of organ dysfunction and who have an excess body fat are labeled as having preclinical obesity.

*The Lancet* group goes further to differentiate obesity−related diseases (diseases in their own right that have causal mechanisms that may be independent of obesity, like cancer) and obesity−induced illness created by obesity−induced dysfunction. See Table 1. Then, they identify 19 obesity−induced diseases (examples include recurrent deep vein thrombosis, male hypogonadism, chronic urinary incontinence, NAFLD (MASLD) with fibrosis, and others) that would qualify an individual for characterization as having clinical obesity. Notably, however, they exclude type 2 diabetes, from among the other common obesity comorbidities. This limits the usefulness of distinguishing preclinical obesity from clinical obesity, since weight management is central to good management of patients with diabetes and most chronic diseases. The Lancet Commission discusses at length whether obesity is a disease or not, detracting from the seriousness of clinical obesity to patients’ health and the health benefits of weight loss in those with preclinical obesity.

**Figure 1:**
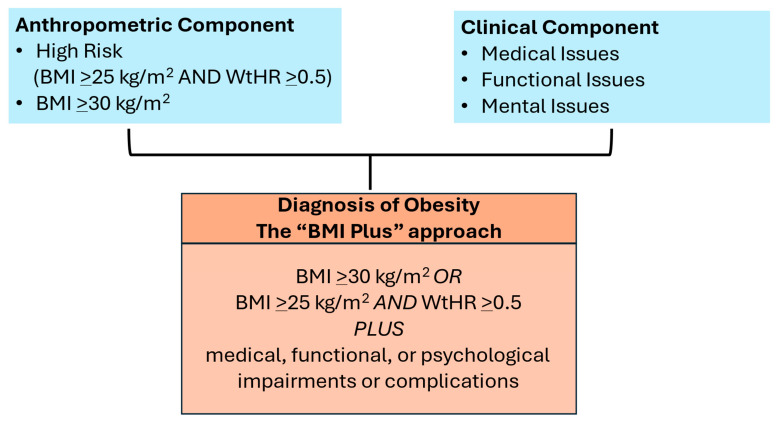
European Association for the Study of Obesity (EASO) framework for diagnosis of obesity (excess body fat).

Another paper from the EASO (European Association for the Study of Obesity) outlined the steps needed to diagnose obesity to initiate treatment with weight management (Figure 1) [2]. Both reports highlight the risks that are associated with obesity when it is accompanied by conditions that may disable the patient. The EASO report maintains the definition of obesity as BMI ≥ 30 kg/m^2^ but adds BMI > 25 kg/m^2^ with a waist−to−height ratio > 0.5, and both criteria require there to be evidence of medical, functional, or mental limitations. This is a simple and actionable approach that maintains some fidelity to the ICD−10 and ICD−11 codes, which are BMI−based and used ubiquitously in medical practice. In contrast, the Lancet Commission approach is to identify individuals with excess fat who may not necessarily be at high risk for ill health and categorize them as “preclinical.” This approach ignores ICD coding, its importance for provider reimbursement of services, and its place as a core measure in the electronic health record. Importantly, both reports emphasize that obesity can drive ill health and can be a disease, and both have raised awareness of the limitations of using BMI alone for a clinical diagnosis to initiate treatment.

In this analysis, we review and provide commentary on the history of clinical efforts to define diagnostic criteria for excess and/or abnormal adipose tissue as the basis for the diagnosis of obesity. Then we ask whether the stigmatizing term obesity is the best one to use to describe the current problem, and what alternatives, if any, there could be. The thesis of this paper is that the time may have come to invest in better ways to diagnose excess and abnormal body fat and to replace obesity with another term, with a focus on healthy weight.

## 2. Adipose Tissue, Weight, and Risk to Health

“Overweight and obesity are defined as abnormal or excessive fat accumulation that presents a risk to health. A body mass index (BMI) is the principal criterion for defining obesity. A BMI above 25 kg/m^2^ is considered overweight, and above 30 kg/m^2^ is obesity. In 2019, an estimated 5 million deaths from noncommunicable disease (NCD) were caused by higher−than−optimal BMI.” [3] (from the World Health Organization)

In 2022, according to the World Health Organization, one in eight people worldwide had obesity, as defined by BMI. This rate has more than doubled since 1990, and obesity in adolescents has quadrupled at the same time. Thus, as of 2022, there were 2.5 billion adults 18 years and older who were overweight and 890 million who had obesity. Of adults aged 18 years and older, 43% were overweight and 16% had obesity. In addition, 37 million children under the age of 5 were overweight in 2022. Among children and adolescents, 390 million children aged 5–19 years were overweight in 2022, and of these 160 million had obesity [3]. The remarkable increase in obesity during this period is not fully explained and is certainly multifactorial. However, conventional wisdom supports the ready availability of energy−dense, highly palatable, highly processed foods high in fat and in refined carbohydrates across the global food supply as contributors.

**Figure 2:**
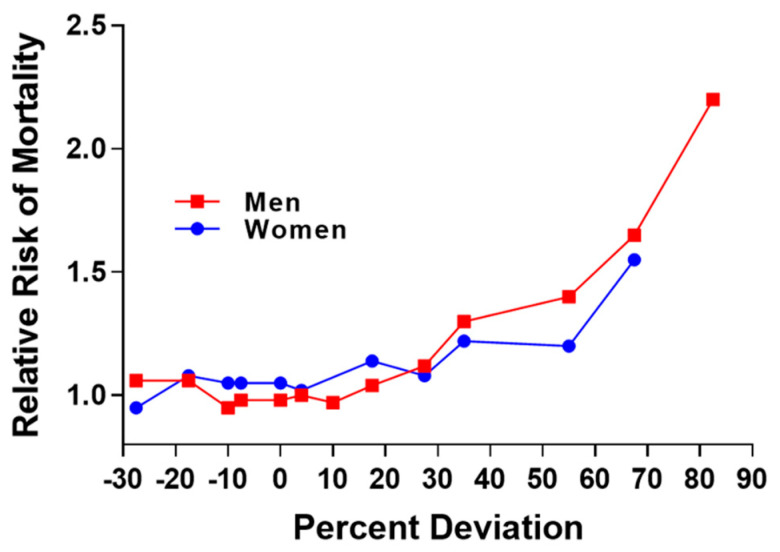
Replication of figure in 1995 publication and derived from actuarial life insurance data from 1913. Relative risk for mortality increases as percent deviation from mean weight for men and women [5].

The relationship between excess fat and disease has been an important concern of the Life Insurance Industry during the 20th century. Initial concerns stemmed from the relation of low or underweight status with risk for tuberculosis and pneumonia [4]. In 1913, the Life Insurance Industry published data showing the curvilinear relation between percent deviation in weight and risk for mortality (Figure 2) and this relationship was illustrated in 1995 [5]. 

This was associated with advice from life insurance companies to maintain a healthy weight and not to gain weight. In 1942−1943, tables of “Ideal Weights” were published by Metropolitan Life Insurance Company (Table 2) [4]. In these tables, weights for both sexes were divided into three groups—small, medium, and large frame size. No clear description of the basis for these frame sizes was provided, although they may reflect the 25th to 75th weight category as a “medium” frame size, with those above and below representing the 5−25th percentile and the 75−95th percentile as the small and large frame sizes, respectively. In 1959, “The Build and Blood Pressure Study” was published, where the weight categories were switched from “Ideal Weight” in the earlier table to “Desirable Weights” in these new tables [6].

## 3. Body Mass Index

The concern that a corporate entity that did not represent all segments of the population was providing information linking body size to health risk led to the introduction of the Fogarty Center Table [4,7] of body weights, which was followed shortly by the body mass index (BMI).

Weight and height are related, and several equations have been developed to express this relationship. BMI is based on the approach proposed by Lambert Adolphe Francois Quetelet (1796–1874), a 19th century Belgian polymath, statistician, and astronomer [8]. The body mass index (BMI) could also be called the “Quetelet Index” after its originator. It is calculated as body weight (kg) divided by the square of height (m^2^) (kg/m^2^). Other relationships of height and weight include Weight/Height; the Ponderal Index (PI) = Weight in kg/Height^3^ (m), which is also known as the Corpulence Index or Rohrer’s Index, Weight (kg) divided by [Height in meters]^3^; weight as a percent of the average weight for that height; and age in relation to body fat measured from body density or the sum of skinfolds.

In a seminal study, Keys and colleagues compared several of these indices to the amount of body fat as estimated from densitometry and as the sum of two skinfolds [9]. The conclusion from this study was that “Judged by the criteria of correlation with height and as a measure of body fatness the ratio of weight to height squared, (The Quetelet index) or the body mass index, is slightly better than the simple ratio of weight to height, and considerably better than the ponderal index”, which was the poorest of the formulas his group studied [9]. The body mass index (BMI) has served as a highly useful estimate of weight relative to height for many years, particularly in studies of populations as opposed to individuals.

The “normal range” of BMI from is 20 to 25 kg/m^2^, a finding that was buttressed by a report in a prestigious British medical journal, showing that among nearly 900,000 individuals, the lowest mortality occurred in BMI range of 22–24 kg/m^2^ [10]. BMI provided a valuable epidemiological tool that delivered the data for recognition in 1992 that the American population had rapidly rising rates of obesity, which has continued to the present [11,12,13,14]. BMI categories for defining obesity vary by age and gender in infants, children, and adolescents, as well as in ethnic groups. Using a sample of almost 1.5 million people from the UK, BMI cutoff point for intervention varied from 30 kg/m^2^ in the UK to 23 kg/m^2^ for Pakistani, Indian, and Nepali South Asians (Table 3) [15]. In addition, most countries identify country−specific cutoff points for BMI to indicate overweight and obesity, as noted in Lancet Commission Appendix 2.5 [1].

Although BMI is valuable for tracking changes in population levels of obesity, it has been criticized for several reasons, one important one being the failure to include fat distribution and another the failure to provide any etiological mechanisms for the development of obesity [16].

But, despite its limitations, BMI appears to be here to stay. The ICD−10 and ICD−11 codes, currently in use around the world, use BMI for the diagnosis of obesity. The ICD codes are required for billing for medical services, and they also have surveillance functions. In the United States, the ICD−10 codes have recently been modified for adults [17] and children [18], but they remain based on determination of BMI. Similarly, ICD−11, used in much of the world, has also been modified [19], but remains based on BMI. The Center for Medicare and Medicaid Services in the US incentivized the inclusion of BMI as one of the Vital Signs in electronic health records, and now BMI is shown at every visit [19].

## 4. Measuring Regional Fat Distribution Changed the View of Body Fat

The dominant concept of obesity in the first half of the 20th century was one put forward by von Noorden in his 1907 chapter on obesity in *Metabolism and Practical Medicine* [20]. Noorden divides obesity into two classes: endogenous obesity including hormonal disorders (secondary obesity) and exogenous obesity resulting mainly from overeating.

The concept of exogenous and endogenous obesity was to be overturned by the concept of regional fat distribution. In 1947, Jean Vague published his first paper on regional fat distribution in *Presse Medicale*, a regional French medical journal. In it he states, “The classical divisions into endogenous and exogenous obesity, those of endocrine origin and those due to overfeeding and insufficient movement, do not provide a key to the problem [of obesity].” His proposal is that “sexual differentiation could throw much−needed new light on the mechanisms of obesity” [21]. Despite the incisive nature of his early papers, the complex way he calculated fat distribution in men and women delayed by 30 years the acceptance of his concept of regional fat distribution as a new biological reality.

Although Vague had published his work in English in 1954 [21], it was not until 30 years later in 1982 that due to the work of Kissebah and his colleagues in the United States [22] and the work of Bjorntorp in Sweden and others [23,24,25,26] that this concept became accepted. Using the circumference of the waist divided by the circumference of the hips, the measure that was used by these investigators showed the power of centrally located fat to predict metabolic diseases.

Several indices for measuring centrally located fat have been compared, including the waist alone, the waist−to−hip ratio, and the waist−to−height (WHtR) measurement. Robust statistical evidence involving more than 300,000 adults in several ethnic groups has shown the superiority of WHtR over waist circumference (WC) and BMI for detecting cardiometabolic risk factors in both sexes. The waist−to−height ratio should therefore be considered as a screening tool [27]. According to the British data, healthy central adiposity is a waist−to−height ratio of 0.4 to 0.49, indicating no increased health risks from abnormal fat distribution. When the value is increased to a waist−to−height ratio between 0.5 and 0.59, this indicates increased health risks from high central adiposity. An even higher value indicated by a waist−to−height ratio of 0.6 indicated the highest risk from centrally located fat [27].

## 5. Limitations to the Term “Obesity”

First, obesity is a pejorative term. In a study of 390 patients with obesity, the least desired terms were “fatness,” “excess fat,” “large size,” “obesity,” and “heaviness,” while the most preferred terms were “weight,” “BMI,” “weight problem,” or “excess weight.” [28] No one wants to be called “obese,” because to the individual it can feel like a judgement of character and lifestyle. As William Banting said in 1863, “Of all the parasites that afflict humanity, I do not know of, nor can I imagine, any more distressing than that of obesity” [29].

Second, obesity is a stigmatized condition. In contrast to such diseases as high blood pressure and heart disease, the presence of obesity is usually visible from the outside, whereas hypertension and high blood pressure require instruments to measure them. The stigmatization of those with obesity begins early in childhood, as demonstated in a famous study of Richardson et al. in 1961 [30]. In this study, children were shown six pictures including a child with no disability, a child with crutches and a brace on one leg, a child in a wheelchair with a blanket over his or her legs, a child with the left hand missing, a child that was disfigured around the left side of the mouth, and a child with obesity. The gender of the pictures was matched to that of the child being questioned. They were then asked to choose the child that would be easiest to play with. That picture was then removed from the list, and the process went on until all children had been selected. The sample included 10− and 11−year−olds from New York, Montana, and Northern California. In all cases, the child with obesity was least likely to be selected as a playmate. Similar differences have been shown in adults.

This seminal work on stigma and obesity was extended by Latner and Stunkard, who showed that things had gotten worse during the 40−year interval between 1961 and 2001. Elementary school children ranked how much they liked children with obesity, various disabilities, or no disability. The children with obesity were ranked lowest in the original 1961 study, yet they were ranked even lower in the 2001 study. Indeed, the difference between the highest−ranked healthy child and the lowest−ranked child with obesity increased by 41% over the four decades [31].

In her excellent chapter on Stigma and Obesity, Puhl summarizes many of the effects of stigma of obesity at different settings during life [32] [Table 4 adapted from Puhl [33]]. The stigma of obesity and associated weight discrimination have been established in virtually all settings, including employment, education, healthcare, media, and interpersonal settings. Of Americans, 19–40% report weight discrimination that increases with the degree of excess weight and is more prevalent in women than men. Workplaces show discrimination based on weight, leading some individuals to down−value themselves. At school, bullying is the most common form of stigma against children with obesity. These experiences at school can lead students to down−value themselves. Many healthcare professionals hold negative attitudes and stereotypes to patients with obesity. Thus, the quality of healthcare can be negatively impacted by the stigma of obesity. Family members, classmates, and healthcare providers are often reported as the most negative influences.

Stunkard had been a major contributor to the literature on stigma and obesity. He and Sobal found a strong inverse relationship between body weight and socio−economic status (SES) among women in developed countries and a positive relationship among women in developing countries [34].

## 6. What Are the Alternatives to the Term “Obesity”?

Over the past 25 years, a number of “guidelines” written by health professionals have provided guidance for the treatment of excess fat or adiposity largely guided by BMI. Some of them are listed in Table 5. Several things stand out from this table. First, BMI plays a key role in the initial decision−making process. Second, the presence of “complications” such as diabetes, cardiovascular disease, sleep apnea, polycystic ovary syndrome (PCOS), MASLD (metabolic dysfunction−associated steatotic liver disease), depression, breathlessness, and osteoarthritis determine the level of response. Lifestyle is the first choice at almost all levels of BMI. Depending on the response and level of BMI, there are many other options, including pharmacotherapy and eventually bariatric surgery.

The word obesity appears in all the guidelines shown in Table 5, but given the negative aspects of the word “obesity”, are there alternatives? Obesity is an increase in the quantity and/or distribution of body fat. Thus, we might use terms that are synonyms for obesity. Since the organ involved in obesity is adipose tissue, “adiposity” would be a leading possibility. The American Association of Clinical Endocrinologists and American College of Endocrinology have suggested that obesity might be called “Adiposity−based Chronic Disease”, or ABCD [42,43]. The European Association for the Study of Obesity has endorsed this concept [44]. This term captures the essence of the process, which begins with the deposition of surplus energy in fat cells as they enlarge, or in younger people replicate, to store the extra fat, which may also be stored in ectopic fat deposits such as visceral fat [45].

**Figure 3:**
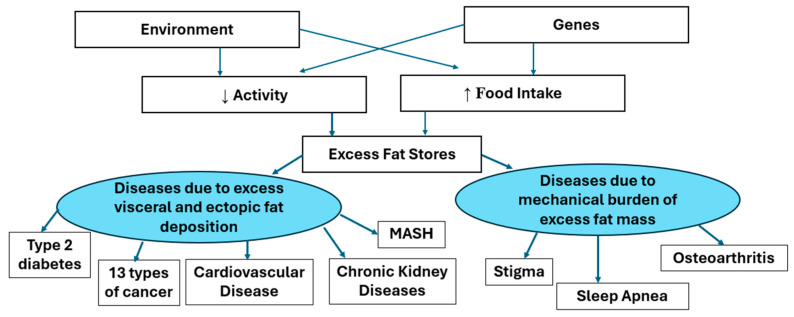
A model for the effects of excess adiposity.

Adiposity−Based Disease focuses attention on the adipocyte and the adipose tissue, which is the origin of the pathology. And the word “adiposity” does not have the stigma associated with the word obesity. The enlarging adipocytes produce adipokines, which have hormonal action on other tissues at a distance. The increasing fat mass can affect other tissues by their mass. Figure 3 below shows the way in which enlarged adipocytes can manifest as other diseases. The AACE and EASO have also provided similar illustrations of the way that body fat and its distribution affect the responses to muscle mass and of the effects of the adipokines released from adipose tissue. Our model shown below is similar by separating the two effects of fat mass and the effects of adipokines into the right and left side of Figure 3.

This diagram shows that genetic and environmental factors mediating increased food intake and/or reduced energy expenditure leading to excess body fat stores. When the capacity of the subcutaneous depots for fat stores is exceeded, fat may be deposited in visceral or other ectopic depots. The mental, functional or metabolic comorbidities associated with obesity (excess adiposity) are caused either by the mechanical burden of excess fat mass or by the products of the enlarged fat mass. 

Current evidence would argue that food intake is more important than changes in energy expenditure in causing obesity. Among 4213 adults from 34 populations across six continents, increased energy intake was roughly 10 times more important than declining total energy expenditure in driving the modern obesity crisis [46]. A meta−analysis of dietary studies shows that consumption of ultra−processed food was associated with increased risk of being overweight and obesity [47].

**Figure 4:**
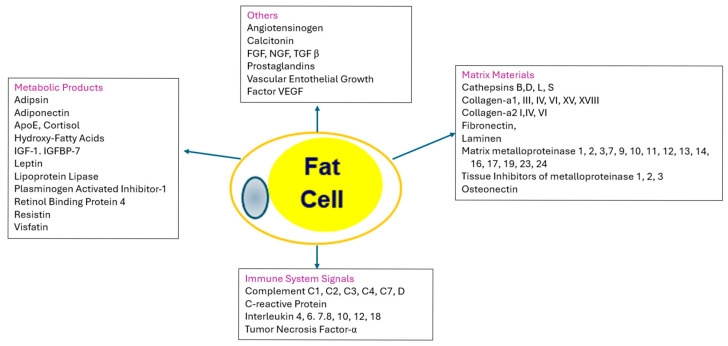
Adipose Tissue as an endocrine organ. The central and left−hand boxes show products that are produced by and secreted by adipocytes. IGF−1 = insulin−like growth factor−1; IGFBP−7 = insulin−like growth hormone binding protein−7; FGF = fibroblast growth factor; TGF β = transforming factor beta; VGVF = vascular endothelial growth factor. The right−hand box contains the proteins that are used for matrix of the tissue: SPARC = secreted protein acidic and rich in cysteine. The bottom box clusters the immune signals from adipose tissue, including various complement proteins and interleukins.

As more fat is stored, the fat cells increase in size and may in some cases increase in cell number. As the capacity to store fat in healthy subcutaneous deposits becomes limited, excess fat may be stored in the visceral (intra−abdominal) compartment or ectopically (in organs such as the liver), giving rise to abnormal adipose tissue functions. The effects of the enlarged adipocytes are manifested in the products or cytokines they produce, their effect on the immune system, and the matrix that holds the adipocytes in place, as shown in Figure 4 [48].

The infiltration of adipose tissue by bone−marrow−derived immune cells that signal via the production of cytokines and chemokines makes the situation even more toxic [48]. However, the primordial mechanism in adults to accommodate excess adiposity is fat cell enlargement with or without the development of insulin resistance.

Since the end of World War II, many new techniques have been added for measuring body fat and adiposity [49]. With magnetic resonance imaging and computed tomography, it is possible to measure total and regional fat with considerable accuracy. Dual−energy X−ray absorptiometry (DXA) allows partitioning of the body into three compartments: fat, muscle, and bone. Hydrostatic weighing or whole−body plethysmography can partition the body into two compartments, fat and non−fat. Bioelectric impedance is used commercially in obesity medicine offices and in home scales and gives a two−compartment model. The technique adds information on body fat percentage but is sometimes inaccurate because of hydration status. Finally, anthropometry, which can be either manual or digital, can provide detailed data about specific dimensions of the individual. Digital anthropometry is as accurate as DEXA in measuring body fat percentage [50] and gives an accurate measure of waist circumference and may overcome the issue of lack of consensus and training on how to measure waist circumference.

With this versatility of methods, why are we focusing so heavily on BMI and waist circumference alone or in relation to height? The first is the ease of obtaining BMI and its versatility in repeated measures of groups of people. A second reason is that data on BMI have become engrained into the regulatory side of managing excess weight. But to overcome the shortcomings of BMI, bringing in measurement of waist will be essential; we believe that digital anthropometry can transform clinical practice. The tape measure has inherent limitations in the clinic, but digital anthropometry requires only a smart phone and can give an accurate assessment of waist circumference and percent body fat [50].

## 7. New Drugs Have Shifted the Treatment Paradigm for Adiposity

One of the important contributions of the Life Insurance Industry in the first half of the 20th century was to note that increased weight was associated with increased risk of mortality, which shortens life expectancy. The idea that identifying health risk from excess body fat could initiate lifestyle changes that would reverse excess body fat through weight loss was the operative idea during much of the 20th century. The futility of long−term weight loss maintenance through lifestyle interventions was a major clinical challenge and for many a disappointment.

The development of drugs targeting Nutrient Stimulated Hormone (NuSH) receptors, such as glucagon−like peptide−1, has ushered in a dramatic change in our approach to weight management. These newer drugs can achieve weight losses of 10%, 20%, or even 30% in significant numbers of patients and return them to a healthy weight. Previously, these weight loss values were only seen after bariatric surgery. At the time of this writing, the FDA has approved liraglutide, semaglutide, and tirzepatide, but there are many more being developed [51].

In addition, these newer drugs have indications not only for weight loss and managing type 2 diabetes, but they can reduce the risk of cardiovascular mortality, combat obstructive sleep apnea, and treat chronic kidney disease. Thus, the imperative today is to accurately identify the individuals whose health risk justifies intervention with these new medications. It is more important now to have an accurate diagnosis.

To sum up, we think maintaining a healthy weight should be each individual’s primary health goal. For all its limitations, BMI is a good guide to initial evaluation. It can remain in the ICD codes as a surveillance measure, perhaps in the Z codes. For a diagnosis of Adiposity−Based Disease (ABD), we favor following the NICE guidelines, which provide the criteria of measuring fat distribution by the Waist/Hip ratio. Whether the best value for Waist/Height is 0.5 is a current area of discussion [52]. Finally, the presence of other evidence of medical, functional, or psychological impairments should be figured into the overall evaluation of patients, which we have performed with the grading system below (Table 6). We might substitute “Adiposity Based Disease” for the term “Clinical Obesity” because it avoids the pejorative and stigmatized term “obesity.”

BMI listed above is appropriate for people of European descent, but as noted earlier in Table 3, there are changes, particularly in the South Asian population, that may lead the user of this table to adjust the initial BMI. Further, we would like to see more efforts at defining a population in whom DEXA body composition assessment makes sense for the diagnosis of regional adiposity and for following potential adverse body composition changes with weight loss. This would seem to be a valid approach in older individuals who may be more susceptible to sarcopenia and bone loss with weight loss.

Our goal in writing this paper was to join the conversation about the best current way to diagnose obesity and to stage the disease to direct treatment. We hope our ideas will stir others to reconsider their thinking on this topic.

## Figures and Tables

**Table 1:** Lancet Commission on conceptualization of preclinical and clinical obesity.

	Preclinical Obesity	Clinical Obesity
Measure: Body Fat Height and Weight Waist Circumference	>25% for males; >30−38% females BMI 25−40 kg/m^2^ Waist/Height <0.5	Same as Preclinical Obesity
Physiopathology	Alterations of cells and tissues leading to alteration of organ structure	Alteration of organ function leading to end organ damage
Clinical Manifestations	Minor or absent; substantially preserved organ function	Signs and symptoms; Limitations in daily activity; Complications
Detection and Diagnosis	Anthropometrics, medical history, Review of Systems, further diagnostic workup as needed	Same as Preclinical Obesity
Treatment approach	Focus on prevention of risk, reduction and progression to clinical obesity or other disease	Focus on improvement or reversal of organ dysfunction

Rubino F, et al. Lancet Diabetes Endocrinol. 2025 Mar;13(3):221−262 [1].

**Table 2:** Life insurance table of ideal weights in 1942−1943.

Woman				Man			
Height (Feet/ Inches)	Weight (lbs)			Height (Feet/ Inches)	Weight (lbs)		
	Frame				Frame		
	Small	Medium	Large		Small	Medium	Large
4′10″	102−111	109−121	118−131	5′2″	128−134	131−141	138−150
4′11″	103−113	111−123	120−134	5′3″	130−136	133−143	140−153
5′0″	104−115	113−126	122−137	5′4″	132−138	135−145	142−156
5′1″	106−118	115−129	125−140	5′5″	134−140	137−148	144−160
5′2″	108−121	118−132	128−143	5′6″	135−142	139−151	146−164
5′3″	111−124	121−135	131−147	5′7″	138−145	142−154	149−168
5′4″	114−127	124−138	134−151	5′8″	140−148	145−157	152−172
5′5″	117−130	127−141	137−155	5′9″	142−151	148−160	155−176
5′6″	120−133	130−144	140−159	5′10″	144−154	151−163	158−180
5′7″	123−136	133−147	143−163	5′11″	146−157	154−166	161−184
5′8″	126−139	136−150	146−167	6′0″	149−160	157−170	164−188
5′9″	129−142	139−153	149−170	6′1″	152−164	160−174	168−192
5′10″	132−145	142−152	152−173	6′2″	155−168	164−178	172−197
5′11″	135−148	145−159	155−176	6′3″	158−172	167−182	176−202
6′0″	138−151	148−162	158−179	6′4″	162−176	171−187	181−207

**Table 3:** BMI cutoff points to trigger action when treating excess adiposity among white and non−white populations.

Ethnicity/Race	BMI Cutoff Point to Trigger Weight Management
White UK (reference population)	30 kg/m^2^
Black	30 kg/m^2^
-Black British	29 kg/m^2^
-Black African	29 kg/m^2^
-Black Caribbean	26 kg/m^2^
-Black other	27 kg/m^2^
Arab and Chinese	27 kg/m^2^
South Asian	24 kg/m^2^
-Pakistani, Indian, and Nepali	24 kg/m^2^
-Tamil and Sri Lankan	23 kg/m^2^
-Bangladeshi	21 kg/m^2^

Adapted from: Caleyachetty R, et al. Lancet Diabetes Endocrinol. 2021 Jul;9(7):419-426 [15].

**Table 4:** Salient Features and Impact of Obesity Stigma.

Prevalence.	Of US adults with obesity, 19-40% report weight discrimination. These rates increase with rising BMI and are of more concern to women than men.
School	Bullying is the most common school stigmatization.
Healthcare	Many healthcare professionals are prejudiced against people with obesity.
Media	Media outlets reinforce negative stereotypes of people with obesity.
Home Environment	Family members contribute to weight-based teasing.
Health Consequences	Stigmatization contributes to depression, anxiety, low self-esteem and lower physical activity; these are documented in childhood, adolescence, and adulthood.
Legal	To date, there are no federal laws that prohibit weight discrimination.

Adapted from Puhl, R. Bias, Discrimination in Obesity. IN: *Handbook of Obesity*. (Eds: Bray GA, Bouchard C. Katzmarzyk PT, Kirwan JM, Redman LM, Schauer PS). Vol 1. Fourth Edition. Boca Raton, FL: CRC Press, Taylor Francis Group, 2024, pp 617-626, 2024 [33].

**Table 5:** Some guidelines for the management of excess adiposity.

Sponsoring Organization, Year, Reference	Recommendations
American Association of Clinical Endocrinologists and American College of Endocrinology 2016 [35]	No complications: lifestyle therapy for all cases. Consider drugs if lifestyle is not enough. If one or more mild-moderate complications;: Add pharmacotherapy if BMI ≥27 kg/m^2^ with complications. If at least one serious complication;: Implement lifestyle and pharmacotherapy and consider bariatric surgery if BMI ≥35 kg/m^2^.
American College of Cardiology, American Heart Association and The Obesity Society 2014 [36]	High intensity comprehensive lifestyle intervention including exercise. Add pharmacotherapy if BMI ≥30 kg/m^2^ or if BMI ≥27 kg/m^2^ with comorbidity. Refer to bariatric surgery if BMI ≥40 kg/m^2^ (or if BMI ≥35 kg/m^2^ with comorbidities.
Obesity Canada 2022 [37]	Nutrition therapy, physical activity, and psychological interventions. Add pharmacotherapy if BMI ≥30 kg/m^2^ (or if BMI ≥27 kg/m^2^ with complications). Consider bariatric surgery if BMI ≥40 kg/m^2^ (or if BMI ≥35 kg/m^2^ with complications).
Australian Guidelines 2022 [38]	BMI 30–40 kg/m^2^ no complications: 10-15% weight loss supervised lifestyle, reduced energy diets or pharmacotherapy. BMI 30–40 kg/m^2^ and complications: 10-15% weight loss with intensive lifestyle, VLED, pharmacotherapy and consider bariatric surgery. BMI >40 kg/m^2^ with complications: >15% weight loss, specialist care, and VLED, pharmacotherapy and bariatric surgery.
Saudi Clinical Practice Guideline 2016 [39]	Lifestyle intervention with individualized counseling. Use metformin or orlistat as preferred pharmacotherapy. Consider bariatric surgery if BMI ≥40 kg/m^2^ (or if BMI ≥35 kg/m^2^ with comorbidities.
European Guidelines 2015 [40]	Nutrition and physical activity with cognitive behavioral therapy. Add pharmacotherapy if BMI ≥30 kg/m^2^ (or if BMI ≥27 kg/m^2^ with comorbidities). Consider bariatric surgery if BMI ≥40 kg/m^2^ (or if BMI ≥35 kg/m^2^ with comorbidities or >30 kg/m^2^ with type 2 diabetes).
NICE Guidelines 2014 [41]	Refer to weight management programs. Provide advice on physical activity, diet quality and energy reduction. Add pharmacotherapy if lifestyle intervention fails. Consider bariatric surgery if BMI ≥40 kg/m^2^ (or if BMI ≥35 kg/m^2^ with comorbidities.

VLED = very−low−energy diet (usually < 800 kcal/d).

**Table 6:** New System for Diagnosis and Staging Adiposity Based Disease.

	BMI	Waist/Height (Measure of Central Adiposity)	Symptoms Related to Excess Adiposity
Healthy weight	<30	<0.5	None
Healthy weight	30 < 35	<0.5	None
Adiposity I	<30, plus	≥0.5 (either or both)	Yes (either or both)
Adiposity II	30 < 40, plus	≥0.5 (either or both)	Yes (either or both)
Adiposity III	≥40	No need to measure	No need to ascertain

“plus” refers to the addition of either or both criteria from column 2 and/or 3.

## Data Availability

There was no original data collected in this work. All sources of data are in the public domain and are cited in the manuscript.
